# Effectiveness of Polyclonal Antibody Immunoconjugate Treatment with Propamidine Isethionate for Amoebic Keratitis in Golden Hamsters

**DOI:** 10.1155/2023/3713368

**Published:** 2023-04-25

**Authors:** Manuel Enrique Ávila-Blanco, Sandra Lizbeth Aguilera-Martínez, Javier Ventura-Juarez, Jorge Pérez-Serrano, Elizabeth Casillas-Casillas, Luis Fernando Barba-Gallardo

**Affiliations:** Universidad Autónoma de Aguascalientes, Aguascalientes, Mexico

## Abstract

*Acanthamoeba griffini* is known to cause amoebic keratitis (AK); its main causes are inadequate hygiene when contact lenses are handled and/or its prolonged use at night, as well as the use of contact lenses during underwater activities. The most used treatment for AK is the combination of propamidine isethionate combined with polyhexamethylene biguanide, which disrupts the cytoplasmic membrane, and damages cellular components and respiratory enzymes. We proposed an immunoconjugate treatment obtained from *Acanthamoeba* immunized rabbit serum combined with propamidine isethionate; the corneas of hamsters inoculated with *A. griffini* (MYP2004) were treated with the combined, at 1, 2, and 3 weeks. Propamidine isethionate is frequently used for AK treatment, *in vivo* study we are found IL-1*β* and IL-10 expression and caspase 3 activity is significantly increased with respect to the group that was inoculated with the amoeba without receiving any treatment, suggesting that it may be an effect of the toxicity of this drug on the corneal tissue. Application of the immunoconjugate showed enhanced amoebicidal and anti-inflammatory activities, with comparison to propamidine isethionate only. The aim of this study is to evaluate the effect of the immunoconjugate of propamidine isethionate and polyclonal antibodies as a treatment of AK in golden hamsters (*Mesocricetus auratus*).

## 1. Introduction

Amoebic keratitis (AK) is a corneal infection caused by the genus *Acanthamoeba*; it belongs to the phylum Amoebozoa [1] and the family of *Acanthamoebidae* [2]. Pathogenic species include *Acanthamoeba griffini*, *Acanthamoeba castellanii*, and *Acanthamoeba culbertsoni*; however, the first report of AK was in 1974 by Naginton et al., caused by *Acanthamoeba polyphaga* [3]. *A. griffini* can cause AK, and its main causes are the lack of hygiene when handling contact lenses and/or their prolonged use at night, as well as the use of contact lenses during aquatic activities [4, 5]; they have also been detected in patients with low levels of Immunoglobulin type A in tears [6]. Nunes Diehl et al. mentioned that this pathology has been reported and has identified 675 cases from 2002 to 2020, of which 253 correspond to Asia, 150 to America, 233 to Europe, and 40 to Africa; being the most frequent genotype T4 (85.92%), followed by T3 (5.92%), *A. griffini belongs* to T3 genotype [7].

Clinically, in AK, there is acute pain, red eyes, limbitis, perineural infiltrates, and punctate keratitis, followed by ring-like infiltrates, epithelial injury, and uveitis [8, 9]*. A. griffini* is in the trophozoite stage, so it can reproduce by binary fission [10, 11]. Acanthamoeba feeds on bacteria, yeasts, and cellular debris via pinocytosis, trogocytosis, and phagocytosis [12–14].

At the onset of damage, amoeba binding to cells occurs via the 133 kDa mannose-binding protein (MPB-133) to membrane glycoproteins of corneal epithelial cells [15–17], leading to the release of proteases, such as MPB eliminate MIP (136 kDa), and induces cell death via activation of phosphatidylinositol-3 kinase [18], triggering the Bak and Bax activation pathways, losing mitochondrial membrane potential, releasing cytochrome c, and activating caspase 3 and mediators of apoptosis [19].

The most used AK treatment is the combination of propamidine isethionate and polyhexamethylene biguanide, which disrupt the cytoplasmic membrane, and damage cellular components and respiratory enzymes [20, 21]. In comparison, diamines, such as 0.1% propamidine isethionate, alter cell membrane structures and permeability, denaturing proteins, and cytoplasmic enzymes [20].

Currently, to improve the efficacy of various immunological treatments, immunoconjugates are used, consisting of immune substances, such as an antibody, which covalently binds to another substance to destroy malignant cells, e.g., a toxin, a radioactive molecule, or a drug [22]. The antibody part of the immunoconjugate we employed *targets A. griffini* trophozoites, and the bound substance affects the membrane of the parasites themselves.

The study aimed to evaluate the effect of the immunoconjugate of propamidine isethionate and polyclonal antibodies as a treatment for AK in golden hamsters (*Mesocricetus auratus*).

## 2. Methodology

### 2.1. Amoeba

*A. griffini* (MYP2004) was characterized by Heredero-Bermejo et al. [23] and donated to the UAA Vision Experimental and Clinical Sciences Laboratory. *Acanthamoeba* was cultured in Cerva medium axenically at 37°C [24] and was used to induce AK during its exponential phase of growth after 36–72 hours.

### 2.2. Amoebic Lysate and Preparation of Rabbit Anti-*A. griffini* Polyclonal Antibodies

*A. griffini* lysate was obtained for the induction of rabbit anti-*A. griffini* polyclonal antibodies according to the methodology of Ventura-Juárez et al. [25].

Titration of New Zealand rabbit anti-*A. griffini* antibody with *A. griffini* antigen was performed by Enzyme-linked immunosorbent assay (ELISA) for detection of trophozoites by indirect immunofluorescence in tissues and cell cultures. Immunoconjugate of rabbit anti-*A. griffini* was prepared to be activated with the propamidine isethionate. To activate propamidine isethionate, 10 ml of Broñlene® (Sanofi, Austria) was taken, and 2 ml of 0.1 M Phosphate-Buffered-Saline (PBS) diluted in 1.25% glutaraldehyde was added and left in agitation for 24 hours at room temperature. 15 mg of rabbit Immunoglobulin type G (IgG) against *A. griffini* at a concentration of 1 : 1000 in PBS, 15 ml of 0.1 M phosphate buffer, and activated propamidine isethionate were used, leaving the mixture for 24 hours at 4°C, to be subsequently dialyzed, lyophilized, and quantified by Bradford's method and antigenic recognition by ELISA.

### 2.3. Animals

Male golden hamsters (*Mesocricetus auratus*), with an average weight of 150 g (±50 g), provided by the Universidad Autónoma de Aguascalientes biotherium, was used. The animals were housed in accordance with the Ethics Committee for the Use of Animals in Teaching and Research of the Universidad Autónoma de Aguascalientes, being compatible with the Official Mexican Norm (NOM-062-ZOO-1999) and consisted of housing five hamsters per box at 20°C and 50% relative humidity with 12-hour light/dark cycles, with food and water available *ad libitum*.

### 2.4. Treatment Groups

Hamsters were divided into 1-, 2- and 3-week groups, each subdivided into five groups (1) non-intervention, (2) saline solution injection (sham), (3) amoeba inoculated, (4) test treatment inoculation (immunoconjugate), and (5) and treatment control (propamidine isethionate). The methodology of Polat et al. was applied, so 7 *μ*L of treatment was administered, in the first week eight times a day (every 2 hours), and for the second and third weeks, the dose is then lowered to three times a day to prevent corneal toxicity [26].

Indirect immunofluorescence microscope protocol for *A. griffini* antibody was titer on. *A. griffini* trophozoites were fixed with 4% paraformaldehyde for 30 minutes at 37°C temperature. New Zealand rabbit serum immunized at 1 : 1, 1 : 10, 1 : 100, 1 : 200, and 1 : 500 concentrations was used, and an Alexa Fluor 488 nm goat anti-rabbit secondary antibody was used as an indirect immunolabel (Life Technologies™, USA).

### 2.5. *In Vivo* Inoculation

Hamsters were nasally anesthetized with 4% sevoflurane in O_2_. The right eye was used as healthy control, and the left eye was used for induction of *A. griffini* keratitis, except for the sham group, who were inoculated with saline solution at 0.9% only. A total of 50,000 trophozoites in a volume of 5 *μ*l resuspended in saline solution at 0.9% were inoculated into each cornea using a 34 G needle and a stereoscope (ZEISS Discovery.V8).

### 2.6. Pathological Analysis and Immunofluorescence

Corneas were fixed in 2.5% paraformaldehyde and processed for embedding in Paraplast® (Sigma, USA) using a histological tissue processor (MICROM STP 120). Hematoxylin and eosin staining was performed according to the methodology of Luna [27], and by immunofluorescence, IL-10 (Abcam, UK), IL-1*β* (Abcam), *A. griffini,* and activated caspase 3 (Cell signaling Technology®, USA) were detected. Alexa Fluor 488 nm goat anti-rabbit Alexa Fluor 488 nm (Life Technologies™) was used as a secondary antibody. The number of positive cells per field for each biomarker was counted.

### 2.7. Pharmacological Drugs and Anesthesia

Propamidine isethionate (Brolene®) was purchased from Sanofi-Aventis (Australia). Sevoflurane 100% (Pisa, Mexico) and pentobarbital sodium was administered ar 10%, 1.5 mL per 100 g of weight intraperitoneally (Laboratorios Aranda, Mexico).

### 2.8. Statistics

An analysis of variance was performed according to the normality test, if the data corresponded to a normal distribution an, Analysis of variance test (ANOVA) was elaborated, lately we include a post hoc analysis; in both tests, a significance level of *α* ≤ 0.05 was considered. The GraphPad Prism software (9.0 version for macOS) was used.

## 3. Results

### 3.1. New Zealand Rabbit Serum IgG Anti-*A. griffini* Antigen

**Figure 1:**
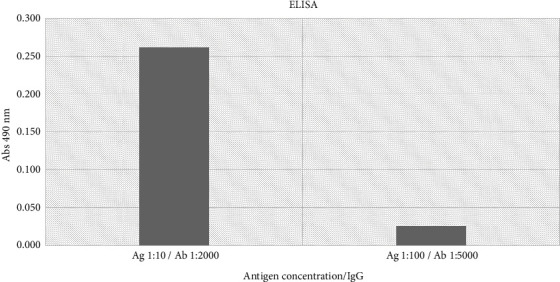
Affinity of New Zealand rabbit IgG against Acanthamoeba griffini amoebic antigen. The affinity was determined using a 1 : 10 dilution of the amoebic antigen and a 1 : 2000 dilution of the IgG.

**Figure 2:**
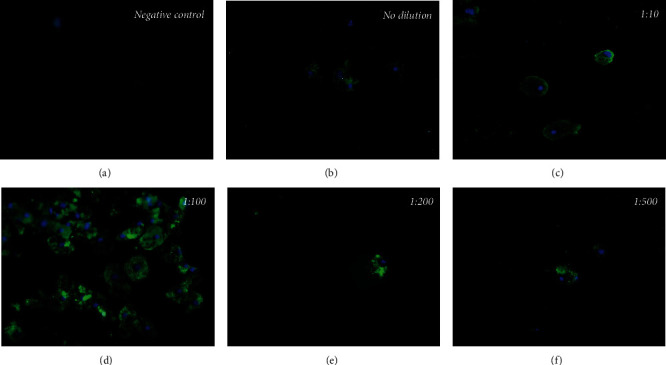
Propamidine isethionate immunoconjugate with New Zealand rabbit IgG. It is observed by immunofluorescence assay that the immunoconjugate recognizes *A. griffini* trophozoites in culture from 1 : 1 to 1 : 500 dilution (×400). (a) negative, (b) no dilution, (c) 1 : 10, (d) 1 : 100, (e) 1 : 200, and (f) 1 : 500.

A New Zealand rabbit antibody immunized with a preparation of *A. griffini* antigen was prepared, blood was collected from the rabbit, IgG was isolated from the obtained serum, and the rabbit antiserum (1 : 2000 dilution) was shown to recognize the amoebic antigen at a concentration of 1 : 10 (Figure 1). The immunoconjugate prepared with New Zealand rabbit IgG with propamidine isethionate is shown to recognize *A. griffini* trophozoites at optimal dilutions of 1 : 2000 by immunofluorescence, applying a second Alexa Fluor 488 anti-rabbit IgG antibody (Figure 2).

### 3.2. Macroscopic Analysis of Keratitis Induction

**Figure 3:**
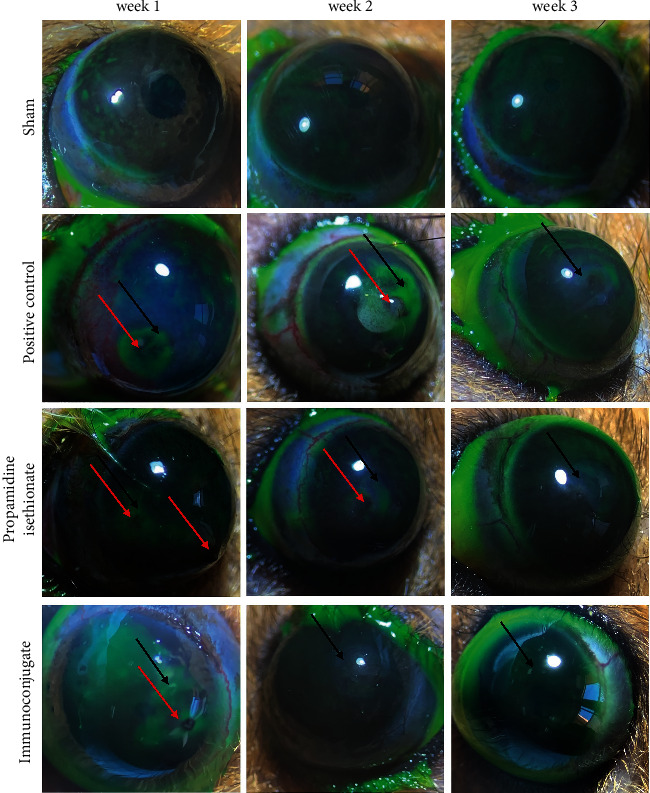
Macroscopic analysis of ulcers. Ulcerative lesions of hamster corneas induced *with A. griffini* are visible in the positive control at 3 weeks after induction. Ulcers were only visible during the first and second weeks of induction in animals treated with propamidine isethionate and are very incipient in the first week in animals treated with immunoconjugate.

The corneas of inoculated hamsters (positive control) with *A. griffini* presented the typical ulceration of amoebic damage at weeks 1 and 2 and in less intensity at week 3 (Figure 3); however, in hamsters that were treated with propamidine isethionate, the ulcers developed presented a smaller area at weeks 1 and 2, not being observed at week 3. Finally, hamsters treated with immunoconjugate-only presented mild lesion formation at week 1 (Figure 3).

### 3.3. Histopathology

**Figure 4:**
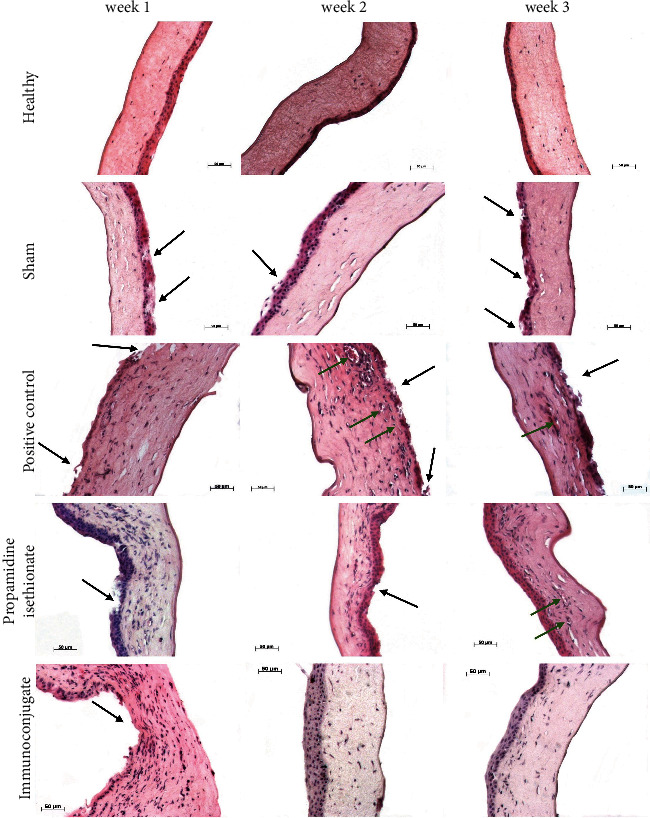
Histopathologic analysis. In inoculated hamsters, ulcerative lesions were observed in the positive control corneal epithelia and associated inflammatory infiltrate in the corneal stroma; however, lesions in animals treated with propamidine isethionate were small with scarce inflammatory infiltrate, and no epithelial lesions or inflammatory infiltrate were observed in immunoconjugate-treated hamsters.

In the corneal samples from healthy hamsters, corneal tissue with preserved histological architecture was observed (Figure 4), whereas in the sham group, some microulcers were observed in the corneal epithelium at weeks 1 and 3, which could have been caused by scratching of the eye by the hamster, this being the only difference between the two groups (Figure 4). However, in the corneas of inoculated and untreated (positive control) hamsters, actual corneal ulcerations developed with the presence of inflammatory tissue in the stroma (Figure 4). In animals treated with propamidine isethionate, only minor ulcerations were observed at weeks 1 and 2, with slight persistence of inflammatory tissue in the stroma (Figure 4); animals treated with immunoconjugate, on the other hand, had only minor ulceration formation in the first week, and no accompanying inflammatory tissue was observed in the following two weeks, showing an appearance of normal corneal tissue (Figure 4).

### 3.4. Immunofluorescence Assay

**Figure 5:**
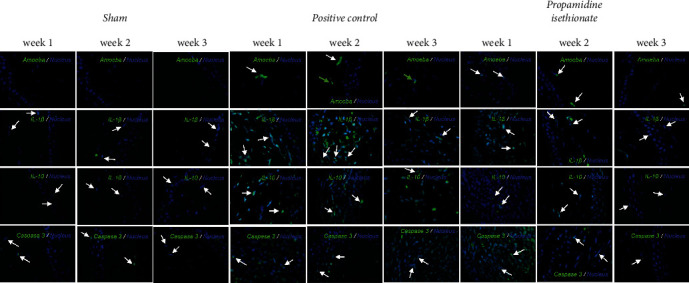
Immunofluorescence for IL-1*β*, IL-10, and caspase 3. No significant differences were found between the healthy and sham groups. Inoculation of the amoeba causes up-regulation of IL-1*β*, IL-10, and caspase 3. Administration of propamidine isethionate causes significant down-regulation of IL-1*β*, IL-10, and activated caspase 3. Immunoconjugate administration significantly reduces cytokine expression. Statistical test was performed according to the normality test, applying ANOVA and Tukey post-hoc test. ∗*p* < 0.05; ∗∗*p* < 0.01; and ∗∗∗*p* < 0.001.

An immunofluorescence assay was performed to identify the presence of amebic, pro-inflammatory, anti-inflammatory, and apoptotic markers in the injured tissue under the different treatments, such as IL-10, IL-1*β*, and activated caspase 3. Based on this, we observed in the control group a basal expression of IL-10, IL-1*β*, and activated caspase 3 (Figure 5); in contrast, we did not observe the presence of *A. griffini* trophozoites or cysts, showing a preserved morphologic architecture in the corneal tissue (Figure 5). However, in the sham group, only a few microulcers were observed in the corneal epithelium, which could have been caused by scratching of the eye by the hamster; this was the only difference between the two groups (Figure 5).

**Figure 6:**
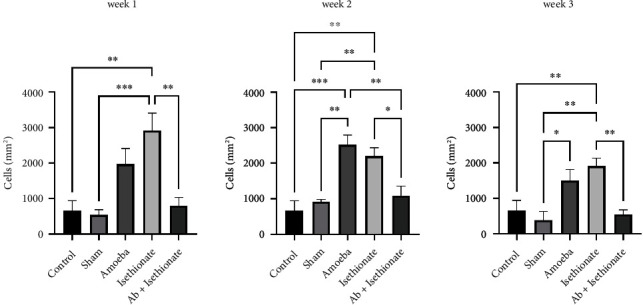
IL-1B positive cells (cells/mm^2^).

**Figure 7:**
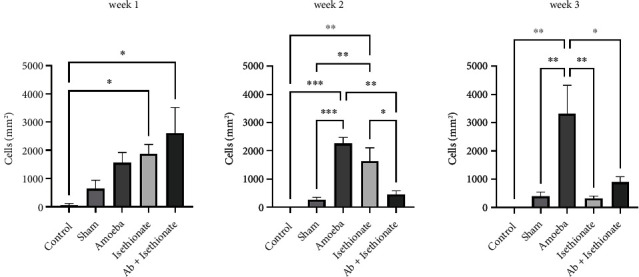
Activated caspase 3 positive cells (cells/mm^2^).

**Figure 8:**
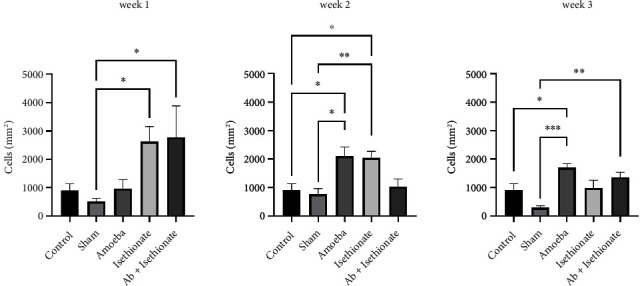
IL-10 positive cells (cells/mm^2^).

Morphological alterations were noted in the positive control group including inflammation, angiogenesis, ulcers, stromal fiber disorganization, and inflammatory infiltrate (Figure 5); an increase in IL-1*β* positive cells (Figures 5 and 6) and caspase 3 positive cells was detected at weeks 1 and 2; at week 3, IL-1*β* positive cells were slightly decreased; in contrast, the presence of caspase 3 positive cells was increased (Figures 5 and 7). IL-10 positive cells were increased at week 2, decreasing at week 3, although it is important to mention that, at week 3, all values in this group have significant differences with respect to the control and sham (Figures 5 and 8). In all tissues, the amoebae were found in the form of trophozoites mainly in the epithelium and in the stroma predominantly in the form of cysts.

On propamidine isethionate administration, caspase 3 positive cells were increased at initial weeks but decreased at week 3 until there was no significant difference from the control. IL-10 and IL-1*β* positive cells diminished over time, with no significant difference at week 3 with respect to the control (Figures 1 and 2). The number of amoebae in the corneal tissue decreased significantly, with mostly cysts being found in the stroma (Figure 5).

**Figure 9:**
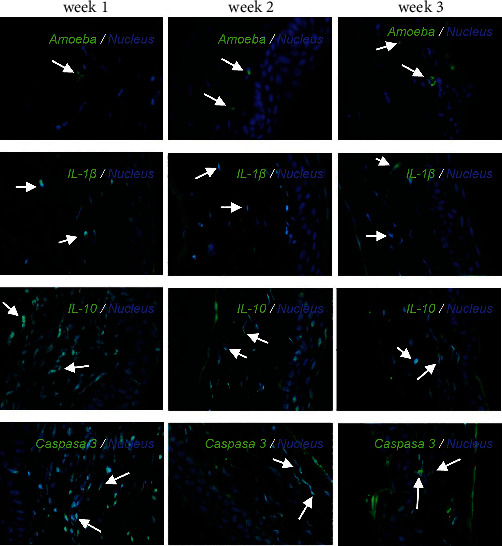
Application of immunoconjugate as treatment against AK.

The immunoconjugate treated in corneas did not present, nor inflammatory infiltrate in the stroma, immunofluorescence assays presented an intact tissue without presence of IL-1*β* positive cells in all weeks of the study, however, if IL-10 positive cells were observable in the first week of the study even higher than the positive control, turning to their control-like state after 2 and 3 weeks of the experiment (Figures 8 and 9).

## 4. Discussion

Effect of proinflammatory, anti-inflammatory, and damage markers *in vivo* under propamidine isethionate treatment, during *Acanthamoeba* keratitis infection, has not been previously characterized. In addition, a polyclonal antibody has not been tested as a treatment for the resolution of AK, making this work to be novel.

IL-10 is an anti-inflammatory cytokine that epithelia constantly express due to recurrent insult from the environment, which results in the release of IL-1*β*, a proinflammatory cytokine. To balance this effect, the cornea releases IL-10 to maintain an anti-inflammatory environment, as it down-regulates the production of IL-1*β* by the epithelial cells, preserving corneal transparency [28, 29]. This accords with our results obtained by immunofluorescence, as there is a basal expression of these interleukins. On the other hand, caspase 3 is a marker of damage that activates the signaling pathway for apoptosis, which in tissues is moderately active to perform tissue remodeling, justifying, as in the case of the healthy and sham groups, the activity of this protein [30]. In sham group, the activation of caspase 3 was increased, which is in agreement with the results of Wilson et al., who report the activation of caspase 3, which initiates the process of apoptosis in corneal tissue during epithelial injury, which, depending on the degree of damage, can be expressed in stromal keratocytes, which in the case of healthy corneas is not often found [30]; according to our results, caspase 3 activity is shown to be decreased at weeks 2 and 3, and the tissue has recovered from the procedure. The desquamation and edema found in the morphological study of the cornea were due to the corneal response to the trauma caused by the puncture and the saline injection [31].

In the corneas of hamsters that were inoculated with Acanthamoeba, similar behavior was shown in IL-10, and IL-1*β* increases at weeks 1 and 2 but decreased slightly at week 3. This pattern of behavior coincided with the research of rat Ávila-Blanco et al. [32] performed in rats, suggesting that it is due to the host immune system intervention. The upregulation of IL-10 is justified by the analyses of Mattana et al. [33], suggesting that the amoeba causes an overexpression of this interleukin to evade the host immune system. However, caspase 3 activity was observed significantly increased at 3 weeks, due to the action of the amoeba on host cells [34]; the damage to the tissue leads to activation of the immune system as an inflammatory infiltrate, and blood vessel development and swelling.

Propamidine isethionate is a drug frequently used for the resolution of AK, for which reason we chose this treatment; yet, there has not been a reported *in vivo* study showing the behavioral kinetics of cytokines and caspases under this treatment through experimental pathology approaches. We found that IL-1*β* and IL-10 expressions and caspase 3 were significantly increased with respect to the group that was inoculated with the amoeba under no treatment; this suggests that it could have been an effect of the corneal tissue toxicity of propamidine isethionate; although a diminution of this biomarker expression was observed as time went by until at week 3 in which there was no significant difference with respect to the healthy and sham controls, this was probably due to tissue remodeling and disease improvement, but there was not a total lesion resolution. On the other hand, there was a considerable decrease of damage in the corneal tissue, although there was still presence of blood vessels, inflammation, and, in some cases, inflammatory infiltrate. Application of the immunoconjugate displayed a higher amoebicide and anti-inflammatory efficacy, with comparison to propamidine isethionate; this result shows the potential of immunological therapies in the treatment of parasitosis as they are already being applied in other fields of medicines, such as breast cancer [22], and in the central nervous system [35]. This allows us to think about other possible more economical and more widely available treatments in combination with the antibodies.

## 5. Conclusion

There is a significant opportunity for an innovative approach to AK treatment through the development of immunoconjugates that can help eliminate *A. griffini* infections and prevent the development of corneal inflammatory states in susceptible individuals.

## Data Availability

The data used to support the finding of this study are available from the corresponding author upon request. The tables of the average number of positive cells with each experimental group used to statistically support the conclusions of this study have been deposited in the DRYAD repository (DOI: 10.5061_dryad.5qfttdz97__v1 (1).zip. Private for Peer Review; https://datadryad.org/stash/share/h3rDJIMiHBkGJRF2kuYrwpr3CTdth4AoDR3xpM0MBqU).
